# LncRNA ANRIL acts as a modular scaffold of WDR5 and HDAC3 complexes and promotes alteration of the vascular smooth muscle cell phenotype

**DOI:** 10.1038/s41419-020-2645-3

**Published:** 2020-06-08

**Authors:** Chengxin Zhang, Shangqing Ge, Wenhui Gong, Jinguo Xu, Zhixiang Guo, Zhuang Liu, Xiaotian Gao, Xiaoyong Wei, Shenglin Ge

**Affiliations:** 1 Department of Cardiovascular Surgery, The First Affiliated Hospital of Anhui Medical University, Hefei, Anhui 230021 China; 2 Department of Rheumatology, The First Affiliated Hospital of Anhui Medical University, Hefei, Anhui 230021 China

**Keywords:** RNAi, Cardiovascular diseases

## Abstract

Many studies have shown that long-noncoding RNA (lncRNA) is associated with cardiovascular disease, but its molecular mechanism is still unclear. In this study, we explored the role of lncRNA ANRIL in ox-LDL-induced phenotypic transition of human aortic smooth muscle cells (HASMC). The results of quantitative fluorescence PCR showed that the expression of ANRIL in patients with coronary atherosclerotic heart disease (CAD) was significantly higher than that in normal subjects. RNA-FISH detection showed that the ANRIL expression increased in HASMC treated by ox-LDL. Ox-LDL could upregulate the expression of ANRIL and ROS and promote the phenotypic transition of HASMC. After downregulation of ANRIL by siRNA, ROS level decreased and HASMC phenotypic transition alleviated. ANRIL could act as a molecular scaffold to promote the binding of WDR5 and HDAC3 to form WDR5 and HDAC3 complexes, they regulated target genes such as NOX1 expression by histone modification, upregulated ROS level and promote HASMC phenotype transition. Therefore, we found a new epigenetic regulatory mechanism for phenotype transition of VSMC, ANRIL was a treatment target of occlusive vascular diseases.

## Introduction

Coronary aortic disease (CAD) represents a series of life-threatening disorders characterized by formation of foam cells and atherosclerotic plaques on aortic wall^1^. Human aortic smooth muscle cell (HASMC) is one of the major components of the medial aortic wall that give rise to a significant number of foam cells and plaques. HASMC is commonly used in studying CAD development and similar coronary diseases in vitro^2^. The phenotypic transformation of HASMCs from contractile state to synthetic state leads to the progression of CAD, and therefore requiring intensive investigation^3,4^.

Oxidized low-density lipoprotein (ox-LDL) is accumulate in atherosclerotic plaques and its presence enhances cell apoptosis of cultured vascular smooth muscle cells (VSMCs), whereas reactive oxygen species (ROS) may modulate the programmed death of VSMCs. Accumulation of ox-LDL was extensively detected in advanced atherosclerotic lesions, as compared with their low expression in healthy arteries^5^. Recent studies have demonstrated that ox-LDL include generation of ROS significantly promoted VSMC senescence^6^. As a response to changes in local environmental cues (such as elevated ox-LDL), HASMCs may switch into synthetic phenotype characterized with increased cell migration, enhanced proliferation rate, and a unique set of synthetic phenotype markers such as osteopontin (OPN) and matrix metalloproteinases (MMPs)^7,8^.

More and more noncoding RNAs as novel modulators are proved to be involved in the regulation of genotype switch in VSMCs and HASMCs^9,10^. Long noncoding RNA (LncRNA) is a new type of noncoding RNAs with a length of >200 nucleotides, which was reported to be abnormally expressed in many diseases including CAD. LncRNAs have showed profound roles in gene expression, which are determined by individual sequences, structure, and biochemistry characteristics^11^. Recently, several lncRNAs were proved to play unexpected roles in regulation of in coronary disease and vascular cell differentiation^12,13^. WDR5 (WD-40 repeat-containing protein 5) is a histone H3K4 presenter that enforces active chromatin structure change and enhanced gene transcription^14^. It was reported that lncRNA bound WDR5 and KAT2A histone acetyltransferase, they acted as a modular scaffold of WDR5 and KAT2A complexes, and coordinated their localization, specified the histone modification pattern on the target genes, and altered gastric cancer cell biology consequently^15^. Histone deacetylase HDAC3 forms a protein complex with several other factors and catalyzes deacetylation in histone tails that mediating gene transcription^16^. HDAC3 and WDR5 were previously found interacting to each other and promoting hypoxia-induced epithelial–mesenchymal transition, suggesting a critical role of WDR5/HDAC3 complex in regulating metastatic phenotypes^17^. Moreover, change of specific chromatin states as mediated by histone marks such as methylation and acetylation on histone lysine sites and other lncRNA modulators are associated with induced phenotype transition^15,18^.

It is well accepted that HDAC3 and WDR5, both bind and recruit the histone-modifying complex to increase active histone marks such as H3K4me3 and activates gene expression. The invlovement between these two as well as lncRNA activities is rarely reported in coronary diseases. In the study, we explored the novel molecular mechanism and implication of a LncRNA antisense noncoding RNA in the INK4 locus (ANRIL). The mechanisms that ANRIL interplays with those histone modifiers and coordinately regulate genotypic switch of HASMC through activated transcription of downstream gene was examined.

## Result

### LncRNA ANRIL expression was elevated in CAD patients and induced by ox-LDL

**Fig. 1:**
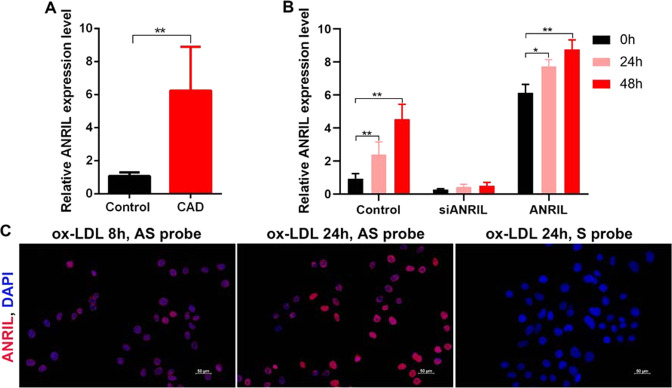
Expression profile of lncRNA ANRIL is enhanced in CAD and induced by ox-LDL. **a** RNA levels of ANRIL in PBMCs from CAD patients and healthy controls. **b**, **c** RNA levels of lncRNA ANRIL in HASMCs treated with ox-LDL, silenced and overexpressed ANRIL measured by qPCR **b** and FISH stating **c**. ANRIL in HA **P < 0.01. Antisense probe (AS) of ANRIL was used in FISH, whereas sense probe (S) was included as negative control. CAD, coronary artery disease; HASMC, human aortic smooth muscle cells; FISH, fluorescence in situ hybridization; ox-LDL, oxidized low-density lipoprotein.

LncRNA ANRIL is a large noncoding RNA previously reported to exist as a genetic susceptibility locus with inverse correlation to coronary disease, type 2 diabetes, and several types of cancer^19–23^. Here, we sought to explore the potential biological role of ANRIL during CAD development. First, the expression profile of ANRIL was measured in PBMCs from CAD patients (*n* = 45) and was found significantly elevated as compared with healthy controls (*n* = 45) (Fig. 1a). Given that ox-LDL has a major role in the formation of foam cells as observed in CAD, we next examined the RNA levels of lncRNA ANRIL in ox-LDL-induced HASMCs. PCR showed that ANRIL RNA levels significantly decreased after siANRIL treatment and they significantly increased after ox-LDL treatment for 8 h, with further induction until 24 h (Fig. 1b). In addition to that, we also performed fluorescence in situ hybridization (FISH) and observed significant induction of ANRIL signal in the cytoplasm of HASMCs after 8 h and 24 h treatment of ox-LDL (Fig. 1c).

### Ox-LDL-induced phenotypic alteration of HASMCs was stimulated by ANRIL

**Fig. 2:**
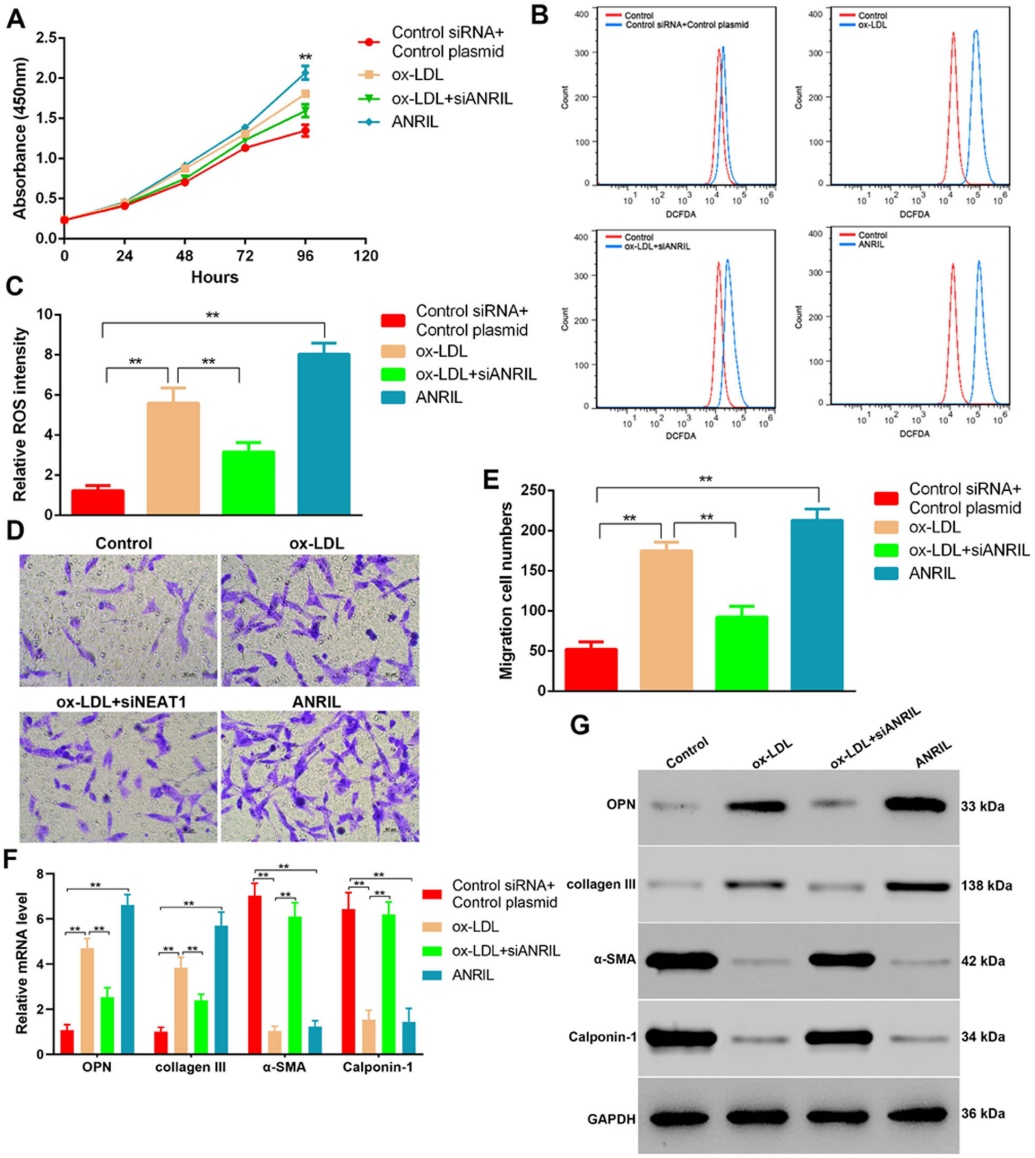
LncRNA ANRIL elicits ox-LDL induced phenotypic change in HASMCs. **a** CCK-8 measured proliferation curves of HASMCs treated by ox-LDL, or transfected with ANRIL overexpressing plasmid and siANRIL. **b** ROS representative histogram images of flow cytometry findings. **c** Cellular ROS levels of HASMCs after treated by ox-LDL, or transfected with ANRIL overexpressing plasmid and siANRIL, as detected by flow cytometry. **d**, **e** Representative images **d** and quantification results **e** of HASMCs with ox-LDL treatment and ANRIL overexpression or silencing, as showed in transwell migration assay. **f**, **g** mRNA **f** and protein **g** levels of OPN, Collagen type III, Cyclophilin 1, and alph-SMA in HASMCs with ox-LDL inducing and ANRIL overexpressing or silencing. CCK-8, cell counting kit-8; ROS, reactive oxygen species.

During ox-LDL-induced plaque formation, smooth muscle cells switch their phenotypes from contractile to synthetic, which is commonly identified by proliferation, migration, and ROS production^24^. Thus, we asked whether ANRIL is involved in the phenotypic alteration of HASMCs. As expected, proliferation assay (Fig. 2a) and ROS synthesis analysis (Fig. 2b, c) showed both ox-LDL and ANRIL overexpression could induce cell growth and ROS production in HASMCs, whereas siRNA-mediated ANRIL repression blocked the cell growth and ROS activation as induced by ox-LDL. Consistent with this, ANRIL silencing markedly reduced ox-LDL promoted HASMCs migration (Fig. 2d, e). Next, we investigated the expression levels of the synthetic phenotype marker OPN Collagen type III, Cyclophilin 1, and α-SMA in these cells. As expected, increase of OPN and Collagen III was induced by ox-LDL and ANRIL and significantly decreased in ANRIL-silenced HASMCs, whereas decrease of Cyclophilin 1 and α-SMA was induced by ox-LDL and ANRIL and significantly increased in ANRIL silenced HASMCs (Fig. 2f, g).

### ANRIL interplays and maintenances HDAC3 and WDR5 interaction

**Fig. 3:**
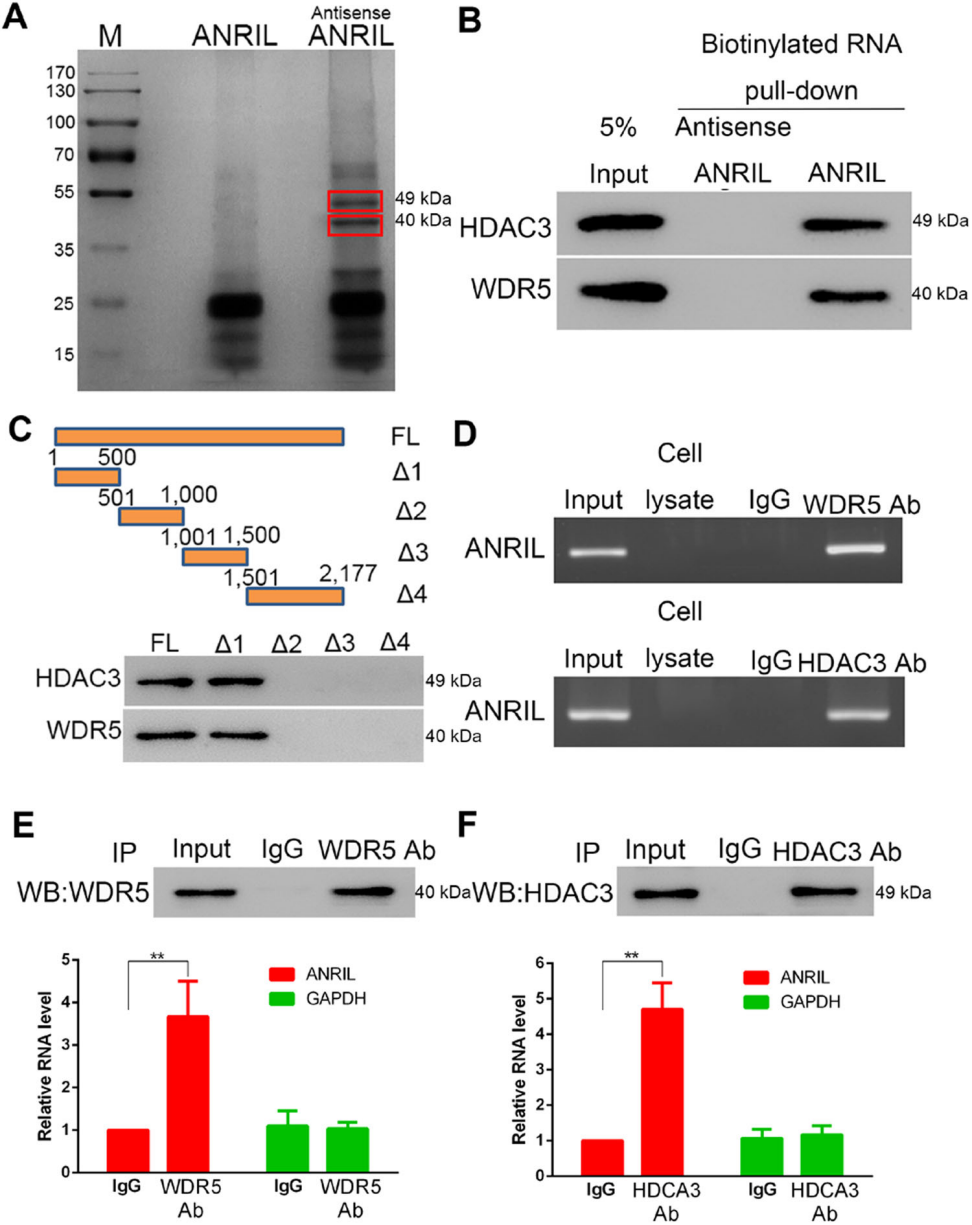
LncRNA ANRIL binds and interplays with HDAC3 and WDR5. **a**, **b** RNA pull-down assay showing the endogenous proteins HDAC3 and WRD5 interact with biotinylated ANRIL in ox-LDL induced HASMCs, as stained by coomassie blue **a** and western blots **b**. **c** RNA pull-down assay to determinate the binding regions of ANRIL with HDAC3 and WRD5 using truncated ANRIL (Δ1: 1–500, Δ2: 501–1000, Δ3: 1001–1500, Δ4: 1501–2177). **d**–**f** Protein immunoprecipitation assay showing ANRIL binds with HDAC3 and WRD5, as pull-downed RNA detected by rapid staining of agarose gels **d** and qPCR **e**, **f**.

Prior reports found histone deacetylase HDAC3 interacts with histone methylation associated protein WDR5, recruiting other the histone modifying complex to regulate chromatin structure and gene transcription as involved in in vitro invasion/migration activity^25^. The potential associations between ANRIL with WDR5 and HDAC3 were investigated by RNA pull-down and RNA immunoprecipitation assays. RNA pull-down results suggested antisense ANRIL significantly pulled-down WDR5 and HDAC3 proteins (Fig. 3a, b) and the 1–500 bp region of ANRIL provided the binding sites to interact with both proteins (Fig. 3c). Protein immunoprecipitation assay detected these RNA–protein interactions through WDR5- and HDAC3-specific antibodies (Fig. 3d). Same results were confirmed by a similar immunoprecipitation assay followed with qPCR targeting ANRIL RNA sequence.

**Fig. 4:**
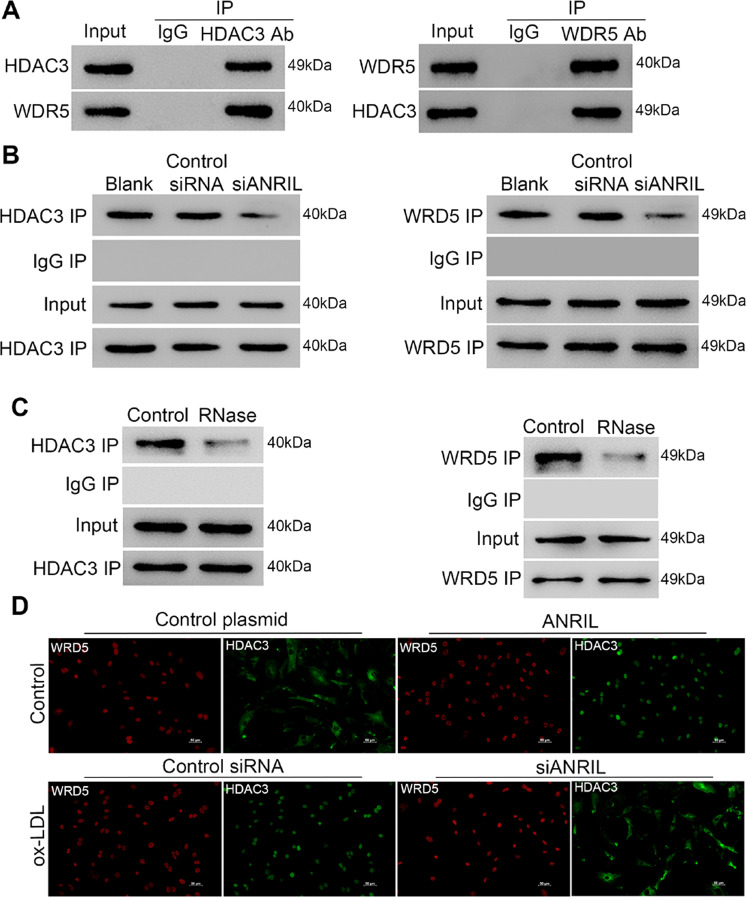
The interaction between HDAC3 and WDR5 requires the presence of ANRIL. **a**–**c** Protein-protein co-immunoprecipitation assay detecting the interaction between HDAC3 and WDR5 in ox-LDL induced HASMCs **a**, ox-LDL induced HASMCs with ANRIL silencing **b** or with RNase treatment **c**. **d** Representative fluorescent immunostaining images of HDAC3 and WD5 in ox-LDL induced HASMCs with ANRIL silencing.

To determine whether the presence of ANRIL is required in WDR5 and HDAC3 protein interactions, protein–protein co-immunoprecipitation assays were performed in HASMCs under various conditions. The results indicated the interaction between endogenous WDR5 and HDAC3 proteins was confirmed in ox-LDL induced HASMCs (Fig. 4a), and was obviously disturbed by treatments of siANRIL and RNase (Fig. 4b, c). Interestingly, immune stating of HDAC3 showed the nuclear translocation of HDAC3 was promoted by overexpression of ANRIL, or stimulation of ox-LDL which was blocked by ANRIL silencing (Fig. 4d).

### ANRIL promotes phenotypic alteration in HASMCs through NOX1 activation

**Fig. 5:**
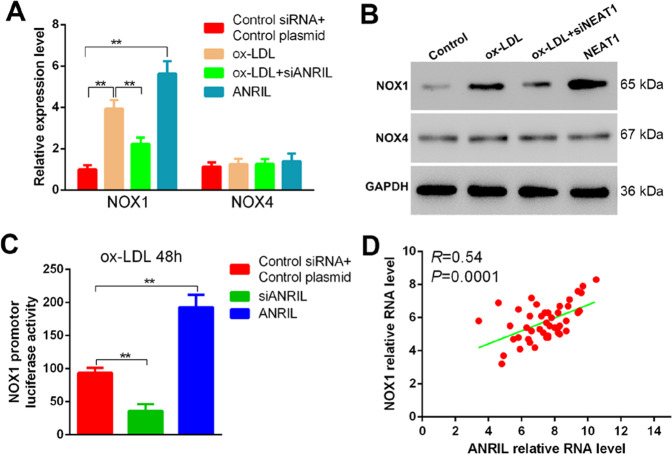
ANRIL binds with NOX1 promoter region and actives its expression. **a**, **b** mRNA **a** and protein **b** levels of NOX1 and NOX4 in HASMCs with ox-LDL inducing and ANRIL overexpressing or silencing. **c** Dual-luciferase activity assay in which ox-LDL induced HASMCs were transfected with NOX1 gene promoter plasmid and ANRIL overexpressing or silencing plasmids. **d** Correction analysis of ANRIL and NOX1 expression levels as measured in PBMCs from CAD subjects (*n* = 45).

NADPH oxidases (NOX) were recently reported to associate with the altered phenotypes in smooth muscle cells^26,27^. To verify whether NOX proteins such as NOX1 and NOX4 are essential in ANRIL promotes phenotypic alteration, siRNA-mediated repression or overexpression ANRIL was performed in HASMCs with or without ox-LDL stimulation. The results showed ANRIL silencing caused a significant loss of NOX1, but not NOX4, in mRNA and protein levels (Fig. 5a, b). The opposite trend was observed in ANRIL overexpressed HASMCs. Moreover, luciferase reporter assay using plasmid containing NOX1 promoter sequence suggested ANRIL promotes NOX1 transcription by interacting to its promoter region (Fig. 5c). Analysis of primary HASMCs from CAD subjects (*n* = 45) suggested a strong correction between expression profiles of NOX1 and ANRIL (Fig. 5d).

**Fig. 6:**
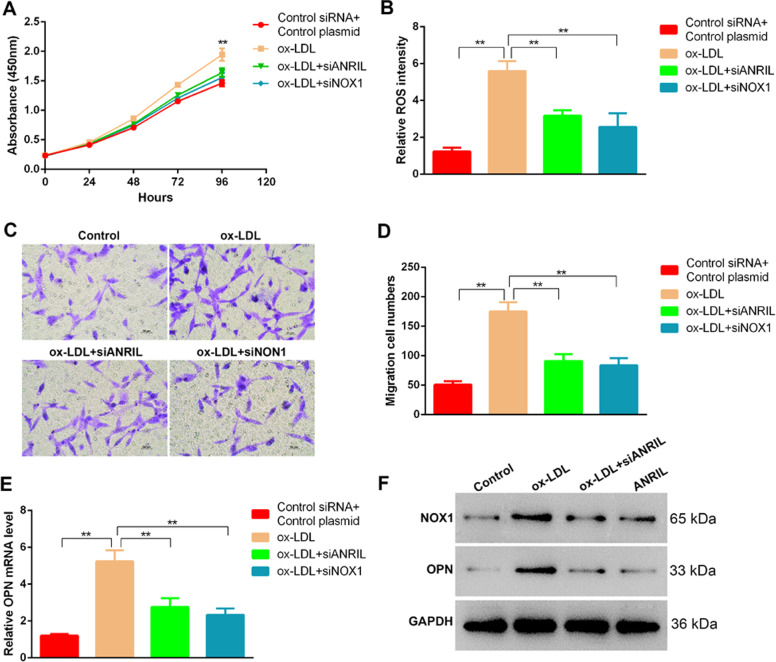
NOX1 silencing blocks ox-LOL-induced phenotypic alteration in HASMCs. **a** CCK-8 measured proliferation curves of HASMCs treated by ox-LDL, or transfected with siANRIL and siNOX1. **b** Cellular ROS levels of HASMCs after by ox-LDL, or transfected with siANRIL and siNOX1, as detected by flow cytometry. **c**, **d** Representative images **c** and quantification results **d** of HASMCs with ox-LDL treatment and ANRIL or NOX1 silencing, as showed in transwell migration assay. **e**, **f** mRNA **e** and protein **f** levels of NOX1 and OPN in HASMCs with ox-LDL inducing and ANRIL or NOX1 silencing.

Next, we asked whether NOX1 is involved in ANRIL promoted phenotype switch in HASMCs. The results showed the enhanced cell proliferation, ROS production and migration activity after ox-LDL treatment were all attenuated significantly by siRNA-mediated repression of NOX1 (Fig. 6a–d). Similar change was observed in expression level of synthetic phenotype marker OPN, together suggesting the critical role of NOX1 to facilitate the phenotype switch of smooth muscle cells of LncRNA regulator ANRIL (Fig. 6e, f).

### ANRIL links WDR5 and HDAC complex and regulates histone marks in gene promoter

**Fig. 7:**
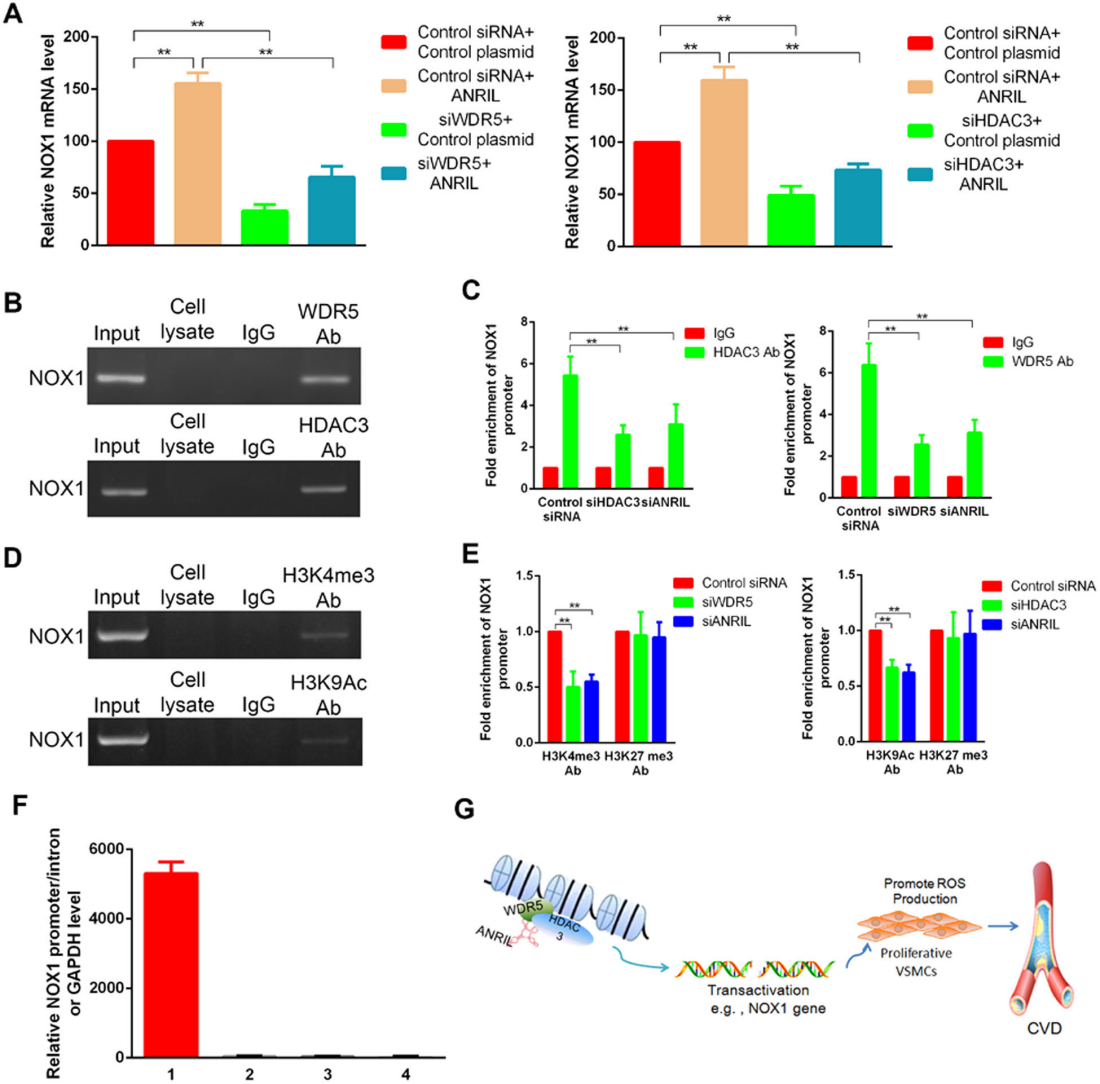
LncRNA ANRIL coordinates the WDR5/HDAC signals and the associated histone marks in NOX1 promoter region. **a** NOX1 mRNA levels in HASMCs that transfected with siWD5 (left) and siHDAC3 (right), with or without ANRIL overexpressing plasmid. **b**, **c** Chromatin immunoprecipitation (ChIP) detecting WD5/HDAC3 binding with NOX1 promoter DNA in ox-LDL induced HASMCs, as pull-downed DNA detected by rapid staining of agarose gels **b** and qPCR **c**. **d** ChIP detecting histone marks (H3K4me3/H3K9ac) binding with NOX1 promoter DNA in ox-LDL induced HASMCs, as pull-downed DNA detected by rapid staining of agarose gels. **e** ChIP detecting histone marks (H3K4me3/H3K9ac//H3K27me3) binding with NOX1 promoter DNA in ox-LDL induced HASMCs with silencing of ANRIL, WDR5, or HDAC3. The pull-downed DNA was detected by qPCR. **f** In vivo interaction between ANRIL with NOX1 promoter was detected in ox-LDL-induced HASMCs by chromatin isolation by RNA purification (ChIRP) assays using DNA antisense oligos(asDNA) specific for ANRIL or a commonly used negative control LacZ. 1, ANRIL asDNA targeting NOX1 promoter region DNA; 2, Negative control LacZ asDNA targeting NOX1 promoter region DNA; 3, ANRIL asDNA targeting GAPDH promoter region DNA; 4, ANRIL asDNA targeting NOX1 intron 1 region DNA. **g** Working model of ANRIL acts a potential modular scaffold interacting with WRD5/HDAC3 and histone marks.

WDR5/HDAC3 complex positively regulates gene transcription with a preference for active histone marks such as H3K4me3 and H3K9ac^16,28^. To examine the effects of ANRIL interacting to the histone modifying complex on the changes of different histone marks in NOX promoter region, chromatin associated analyzes were performed. In HASMCs overexpressing ANRIL, the repression of both WDR5 and HDAC3 significantly reduced NOX1 expression (Fig. 7a). In consistent with that, strong bindings of WD5/HDAC3 and active marksH3K4me3/H3K9ac on NOX1 promoter were detected by a series of chromatin immunoprecipitations (Fig. 7b, d). Notably, significant decrease of these binding signals was observed in ANRIL-silenced cells (Fig. 7c, e), suggesting ANRIL coordinates the localization of WDR5/HDAC3 and regulates the associated epigenetic marks in downstream promoter element. The repressive mark H3K27me3 served as a control here and showed no significant change. In addition, the specific in vivo binding between ANRIL RNA with NOX1 promoter DNA was confirmed by chromatin isolation by RNA purification (ChIRP) assay, whereas no RNA-DNA binding was detected between ANRIL with NOX1 intron DNA, promoter DNA of control gene GAPDH or a control RNA LacZ with NOX1 promoter DNA (Fig. 7f).

## Discussion

Although emerging evidences provided improved understanding of lncRNA function and its molecular mechanism in cellular response and disease development, the mechanisms governing the multi-activities of lncRNA ANRIL remain poorly understood. Here, we performed a comprehensive study aiming to dissect the critical role of ANRIL during CAD development and HASMC synthetic phenotype transformation. Our results demonstrated ANRIL serves as a potential scaffold protein not only interacts with WDR5 and HDAC3 histone modifying complex but also affects epigenetic regulatory signals on NOX1 promoter region (Fig. 7g). Our results improved understanding of the mechanism and molecular processes underlying the ox-LDL-induced phenotypic change of HASMCs, known to have an essential role in the development of progression of CAD.

Prior studies have reported the close association of ARNIL with atherosclerosis-related diseases. For example, the different SNPs in ARNIL was found that be a risk factor for atherosclerotic vascular diseases and was linked to the inflammation regulation, which is a major cause of CAD^29^. Genome-wide association studies have identified chromosome 9p21 (Chr9p21) locus as a susceptibility locus of CAD^30^. Actually, ARNIL transcripts was found adjacent to Chr9p21 and the genetic variation as well as its expression level was directly correlated with severity of atherosclerosis^31,32^. To directly assess the clinical relevance of ARNIL in coronary disease and atherosclerotic plaque formation, here we investigated the mRNA profiles of ARNIL in PBMCs collected from CAD subjects as well as in the ox-LDL-induced HASMCs. We observed a significant increase of ARNIL levels in CAD PBMCs, indicating a strong association of ARNIL in blood cells with CAD development and progression. Our result also demonstrated that ox-LDL treatment significantly promoted ANRIL expression in HASMCs. These results together are supported by a previous study in rat model with coronary atherosclerosis. The rat study reported the serum levels of circular ANRIL, LDL protein, total cholesterol, and several inflammation factors such as IL-1 and IL-6 were elevated in the ANRIL overexpressed rats^30^.

During the development of plaque formation, HASMCs show loss of contractility and acquisition and exhibit a wide range of synthetic phenotypes, including increased cell proliferation, migration, and ROS production. Prior reports regarding to the cellular function of ANRIL were majorly focused in cancers. Generally, enhanced ANRIL expression was identified in many cancer types including gliomas, colorectal cancer, cervical cancer as well as hepatocellular cancer. Suppression or knockdown of ANRIL resulted in inhibiting the cancer cell proliferation, migration, and invasion^33–36^. To date, very rare study showed the cellular function of ANRIL in aortic smooth muscle cells. The previous study of rats with coronary atherosclerosis found reduced ANRIL could prevent coronary atherosclerosis by reducing apoptosis of vascular endothelial cells^30^. These studies together are in consistent with our findings that ANRIL promotes cell growth and migration in HASMCs. Our study also characterized that ANRIL promotes generation of ROS, which is known to trigger phenotypic switch of vascular cells and lead to lesion formation via influencing dysfunction in vascular cells^37,38^. Osteopontin secretion is one of the most classical events in vascular cells that are undergoing pathologies phenotypic modulations^39^. Our study observed ANRIL significantly induced OPN in both mRNA and protein levels, providing another strong evidence that this lncRNA triggers phenotypic switch and pathologic change in HASMCs.

Recent studies have discovered a large number of genomic binding sites in noncoding RNAs, contributing to the lncRNAs-mediated regulation of chromatin structures^40^. Our study here demonstrated ANRIL, as another DNA-binding lncRNA, provides binding sites to promoter region to its downstream gene NOX1, and helps facilitate interactions with chromatin-modifying proteins HDAC3 and WDR5. In support to our findings, prior reports showed circular ANRIL binds to a ribosomal assembly factor pescadillo homolog 1 in vascular cells, which consequently impairs the exonuclease-mediated ribosomal RNA processing^30^. Another example is ANRIL directly recruits polycomb proteins in at least two distinct forms, PRC1 and PRC2 protein complexes, to modify the epigenetic marks in histone and thus mediate hundreds of genes in cis-regulation^29^.

NADPH oxidases have a central role in the pathogenesis of cardiovascular disease. Although both NOX1 and NOX4 were previously elucidated to associate with the altered mechanism in dysfunction of smooth muscle cells^26,27^, our findings seems the first report of a novel lncRNA interacting with NOX1 but not with NOX4. The switched phenotypes as measured in HASMCs were remarkably attenuated after knockdown of NOX1, suggesting ANRIL basically promotes the phenotypic alteration through sequence-specifically binding and regulating on NOX1 gene. Our study thus added new evidence that transcriptional regulation of NOX1 are involved in ox-LDL induced ROS production and cell migration in vascular cells, suggestingNOX1might be targeted for the purpose of treating coronary diseases.

Interestingly, to date only one prior study have recognized the direct interplay between HDAC3 and WDR5, stating they interact to each other and serve a key molecular procedure that coordinately regulates hypoxia-induced epithelial–mesenchymal transition^16^. In response to hypoxia stimulation, histone deacetylase HDAC3 interacts with histone H3K4 methylation presenter WDR5, and together recruits the histonemethyltransferase complex to increase active histone marks in chromatin, and activates mesenchymal gene expression. In agree with the prior report, we observed direct binding of WDR5 and HDAC3, which was recognized by lncRNA ANRIL. We also demonstrated and the roles of WDR5/HDAC3 in the regulation of ox-LDL induced NOX1 activation and the phenotype switch in HASMCs. On top of that, our study on lncRNA added a new layer to this the existing working model of HDAC3/WDR5 and H3K4me3/H3K9ac. Actually, a very recent study reported that a lncRNA named ANRIL acted as a modular scaffold of WDR5 together with a histone acetyltransferase KAT2A, coordinated their localization and targeting on histone modification pattern on the downstream genes^17^. Similarly, here we propose a new working model of HDAC3/WDR5, in which ANRIL as an ox-LDL responsive lncRNA does not only binds to HDAC3 and WDR5 individually, but also drives the nuclear translocation of HDAC3 in vascular cells and guides the binding of this complex to promoter region. As a conclusion, our results suggested that ANRIL serves as a potential modular scaffold facilitating the activator function of HDAC3/WDR5 in HASMCs.

This study needs further exploration to overcome a few limitations. Such as visualization of synthetic HASMCs in tissue structure and interaction of lncRNA with histone marks in subcellular levels could be performed using animal models through IHC and FISH, which certainly needs to be verified in the future.

In summary, of the diverse findings of all the putative molecular mechanisms assigned to lncRNA, our study successfully tested a new hypothesis and consequently provided a new insight into the interplay between lncRNAs and epigenetic machinery that involved in the modulation of chromatin conformation. Although the interaction of lncRNAs with other RNA, DNA, and protein as in regulating gene expression or controlling chromatin structure have been frequently highlighted in carcinogenesis and stem cell development^41^, its involvement in other cellular functions such as vascular cell phenotypic changes was not clear. Drawing new connections between the discoveries will evidently contribute to our ever-improving understanding of how atherosclerosis and other coronary diseases were developed and whether novel lncRNA-targeted medicine could control the biological outputs during foam cell formation in atherosclerotic plaques.

## Materials and methods

### Human peripheral blood samples

Peripheral blood samples from 45 patients with coronary atherosclerosis were collected from the Vascular Surgery Department, First Affiliated Hospital of Anhui Medical University from January to December 2017. Forty-five control peripheral blood samples were from healthy people in the physical examination center, the First Affiliated Hospital of Anhui Medical University.

Informed consent was obtained from all subjects and that the experiments conformed to the principles set out in the WMA Declaration of Helsinki and the Department of Health and Human Services Belmont Report. The Ethics Committee of Anhui Medical University approved this study.

### Cell culture and siRNA transfection

HA-VSMCs are purchased from ATCC. Cells were cultured using medium DMEM/F12 with 10% FBS, 1% penicillin, and 1% streptomycin (Gibco, Thermo Fisher Scientific, Inc., Waltham, MA, USA) at 37˚C with 5% CO_2_. The siRNA and pCNDA3.0-ANRIL transfection were performed with Lipofectamine 2000 kit according to the kit’s manual (Invitrogen, Thermo Fisher Scientific, Inc.). The ANRIL and NOX1 siRNA were transfected into HA-VSMCs, respectively. In ox-LDL treatment group, ox-LDL (50 μg/ml) was added into the medium after transfection.

### Quantitative real time -PCR (qRT-PCR) analysis

Total RNA was extracted by a Trizol reagent kit (Thermo Fisher Scientific, Inc., Waltham, MA, USA). The purity and concentration of RNA were detected by a NanoDrop 1000 Spectrophotometer. RNA (1 μg) was subjected to reverse transcription with a MMLV Reverse Transcriptase Kit. qPCR was performed by a PCR amplification kit (Qiagen China Co., Ltd., Shanghai, China) with ABI StepOne Plus system. They were quantified by the 2^−ΔΔCT^ method. The thermocycling condition was: 95 °C for 10 min, followed by 40 cycles of 95 °C for 10 sec and 60 °C for 40 sec. The internal control was GAPDH gene. The used primers are shown in Table 1.

**Table 1:** Sequence of primers used in this study.

Gene	Forward primer (5′–3′)	Reverse primer (5′–3′)
ANRIL	GCCTCATTCTGATTCAACA	TAGAAAGCAGTACTGACTCGG
OPN	TGAAATTCATGGCTATGGAA	TGAAACGAGTCAGCTGGATG
NOX1	TTGTTTGGTTAGGGCTGAATGT	GCCAATGTTGACCCAAGGATTTT
NOX4	TGTGCCGAACACTCTTGGC	ACATGCACGCCTGAGAAAATA
Collagen III	CTTCCTACGGGGAATCTGTGT	CAATGGCGTTTTGGGTGTTC
α-SMA	AGGTAACGAGTCAGAGCTTTGGC	CTCTCTGTCCACCTTCCAGCAG
Calponin 1	CTGTCAGCCGAGGTTAAGAAC	GAGGCCGTCCATGAAGTTGTT
GAPDH	GCTCTCTGCTCCTCCTGTTC	ACGACCAAATCCGTTGACTC

### RNA-FISH

Sense or antisense RNA probes labeled by digoxigenin were synthesized using MAXIscript T3 and T7 in vitro transcription kit (Thermo Fisher Scientific, Inc., Waltham, MA, USA) with the T3 and T7 promoters in the blunt vector. FISH assay were performed essentially following the published protocol. In brief, cells were harvested and fixed with 4% paraformaldehyde, they were permeabilized in acetone and methanol solution (1:1), and then they were hybridized with ANRIL antisense/sense RNA probes. The hybridized sections were incubated with antibody (anti-digoxigenin antibody conjugated by peroxidase) after peroxidase quenching and blocking and revealed using SuperGloTM Green Immunofluorescence Amplification Kit (Fluorescent Solutions, Augusta GA, USA). Nuclei were counter-stained with DAPI. Images were taken using the confocal microscopy (LSM780 upright, Zeiss).

### Immunofluorescence

Cells were harvested and fixed in 4% paraformaldehyde. They were cut into sections and washed with PBS. After 30 min of blocking with goat serum (10%), sections were incubated using anti SM α-actin (mouse, 1:500) antibodies. The secondary antibody (568 nm anti-mouse secondary antibody, 1:250 dilution, Invitrogen) diluted with blocking buffer were used to stain sections at room temperature for 1 h. Sections were mounted with mounting medium to visualize nuclei. Images after stained with fluorescence-labeled secondary antibody were collected using confocal microscopy (LSM780 upright, Zeiss) as described above.

### Protein extraction and western blotting

Proteins were extracted from HCASMCs as previously described. In brief, after rinsed with PBS, cells were harvested and proteins were extracted using radioimmunoprecipitation assay buffer. Proteins were quantified by bicinchoninic acid assay after sonication and centrifugation. They were loaded in sodium dodecyl sulfate-polyacrylamide gel electrophoresis gel (6–9%, 5–10 μg per lane). Antibodies Osteopontin (abcam, 1:500), Collagen III (abcam, 1:500), α-SMA (abcam, 1:500), Calponin 1 (abcam, 1:500), HDAC3 (abcam, 1:500), WDR5 (abcam, 1:1000), NADPH (abcam, 1:1000), NOX1 (abcam, 1:1000), GAPDH (abcam, 1:2000) were used in this study. Images were collected by ImageQuant LAS 4000 Imaging Station (GE), the densities of bands were quantified with the ImageQuant TL software (GE).

### RNA pull-down assay

Biotinylated ANRIL was refolded in NEB enzyme buffer with RNase-out (Invitrogen, USA) at a final concentration (200 ng/μL). The diluted RNAs were incubated at 60 °C for 10 min and slowly cooled to 4 °C. Aliquots (2 μg) of folded RNAs were used for pull-down experiments. To prepare cell lysates, BGC823 cells were harvested using 5 mL of buffer A (10 mM Tris·HCl, pH 7.0, 1.5 mM MgCl_2_, 10 mM KCl, 0.5 mM DTT, 1 mM PMSF, and protease inhibitor mixture). Cells were lysed by the addition of 0.25% Nonidet P-40 and incubated for 10 min at 4 °C. The lysates were centrifuged at 2500 × *g* for 15 min, and the supernatant was discarded. Pellets containing the nuclear fractions were re-suspended in 3 mL of buffer C (25 mM Tris·HCl, pH 7.0, 0.5% Nonidet P-40, 150 mM KCl, 0.5 mM DTT, and protease inhibitor mixture) and sheared by homogenizing for 15–20 strokes. Samples were centrifuged at 15,000 × *g* for 10 min. The concentration of proteins in the nuclear lysates was measured by the DC assay (Bio-Rad, USA). For the pull-down incubations, nuclear lysates were precleared with streptavidin beads, they were incubated with 2 μg of biotinylated RNA and 40 μl of streptavidin beads at 4 °C for 2 h. After centrifugation, beads were collected and washed with buffer C for three times. RNA-associated proteins were stained by silver, and then they were eluted and resolved by SDS/PAGE.

### Database search, LC-MS analysis, and protein identification

ANRIL and Antisense ANRIL pulled-down eluates were compared with identify-specific ANRIL interactors. The bands predominantly represented only in the ANRIL pulled-down sample were chosen. They were excised to perform in-gel trypsin digestion and peptide extraction. In brief, coomassie brilliant blue dye on gel slices were removed with acetonitrile (50%, ACN)/ammonium bicarbonate (50 mM), they were dehydrated in CAN (100%). Then gel slices were reconstituted at 37 °C overnight using an in-gel digestion buffer containing 12.5 ng/μl trypsin for protein digestion. Extract the tryptic peptides from the gel pieces using 50% ACN/0.1% trifluoroacetic acid (TFA), the tryptic peptides were lyophilized. LC-MS experiment was conducted on a nano Acquity UPLC system, which was connected to a LTQ Orbitrap XL mass spectrometer equipped with an online nano-electrospray ion source according to the instructions. The spectrum was recorded by Xcalibur software. The mass spectra generated by the LTQ-XL instrument were processed with MaxQuant software (http://www.maxquant.org/). The data were searched by Andromeda search engine in human UniProtKB/Swiss-Prot database (Release 2012_12_07, 20233 entries). The search parameters of the database were set as follows: (1) the minimum required peptide length was seven amino acids. (2) Trypsinase splitting specificity applies to splitting that allows up to two deletions. (3) Variable modifications contained methionine deamidation (NQ) and oxidation (M). (4) The mass tolerance for fragment ions and precursor was set to 0.5 Da and 10 ppm, respectively. (5) Both the peptide and protein levels, the false discovery rate was set to 1%. (6) It was considered a reliable identification only when proteins sequenced with at least two peptides.

### RNA immunoprecipitation

RNA immunoprecipitation (RIP) detections were conducted with the Megna RIP Kit (Millipore, USA). The co-precipitated RNAs were measured by RT-PCR. Total RNA (input controls) and normal rabbit IgG controls were simultaneously assayed to demonstrate that the detected RNA signals specifically bind to WDR5 or HDAC3. The primers used for ANRIL are in table 1.

### Co-immunoprecipitation

Co-immunoprecipitation was conducted as ref. ^18^. Both input samples and IP samples were analyzed by western blotting method.

### ChIRP

Use the online probe designer (singlemoleculefish.com) to design ANRIL asDNA (antisense DNA), ANRIL sDNA (sense DNA), and LacZ asDNA (antisense DNA) probes. Oligonucleotides were biotinylated at the 3′ end with an 18-carbon spacer arm. ChIRP was performed as the reference described^42^.

### Transwell and ROS assay

Transwell test was performed with 24-well transwell plates (8-μm pore size), which were precoated with Matrigel. Cells were harvested and seeded (1 × 10^5^ cells) in medium (serum-free) into the upper chamber, whereas medium containing 20% FBS was applied to the lower chamber. The migrated cells were fixed, stained, and counted after incubation for 48 h.

ROS accumulation was determined by the fluorescent probes 2’, 7’-dichlorodihydrofluorescein diacetate (H2DCFDA). Cells were stained using the H2DCFDA fluorescent dye for 10 min. They were trypsinized and re-suspended in PBS. Fluorescence was detected at specific time intervals by a flow cytometer.

### Luciferase assay

The vectors expressing the designated combinations of pGL3-NOX1P WT and other relevant siRNAs were transfected into cell lines at 1.0 μg and 100 ng of phRL (Renilla Luciferase) using Lipofectamine 2000/DharmaFECT 1 transfection kits. The cells were collected and the luciferase activity was detected by the Dual-Luciferase reporter system after transfection for 24 hours. BD Monolight 3010 luminometer was used to measure Luciferase activity. The efficiency of transfection was normalized using the corresponding Renilla luciferase activity.

### Statistical analysis

The SPSS 17.0.1 software was used to carry out statistical analysis. Data are presented as the means±standard error (SE) or standard deviation (SD). Whether the data were normally distributed was tested using the One-Sample Kolmogorov–Smirnor test. The measurement data between two groups were compared using the paired-sample *t* test or independent-sample *t* test if they were normally distributed and the variation was comparable. One-way analysis of variance test were firstly performed among three or more groups if the variation were comparable. If the results showed significant difference, the difference between the two groups was compared with the Student Newman Keuls analysis. When the data were shown the skewed distribution, comparisons were performed by nonparametric tests. Enumeration data were examined by Chi-square test or Fisher Exact test. The correlation of the two genes was examined by Spearman correlation test. It was considered significant with a value of *P* < 0.05.
